# Expression of Calmodulin and Myosin Light Chain Kinase during Larval Settlement of the Barnacle *Balanus amphitrite*

**DOI:** 10.1371/journal.pone.0031337

**Published:** 2012-02-13

**Authors:** Zhang-Fan Chen, Hao Wang, Kiyotaka Matsumura, Pei-Yuan Qian

**Affiliations:** KAUST Global Collaborative Research Program, Division of Life Science, The Hong Kong University of Science and Technology, Hong Kong SAR, China; University of Rome, Italy

## Abstract

Barnacles are one of the most common organisms in intertidal areas. Their life cycle includes seven free-swimming larval stages and sessile juvenile and adult stages. The transition from the swimming to the sessile stages, referred to as larval settlement, is crucial for their survivor success and subsequent population distribution. In this study, we focused on the involvement of calmodulin (CaM) and its binding proteins in the larval settlement of the barnacle, *Balanus ( = Amphibalanus) amphitrite*. The full length of *CaM* gene was cloned from stage II nauplii of *B. amphitrite* (referred to as *Ba-CaM*), encoding 149 amino acid residues that share a high similarity with published CaMs in other organisms. Quantitative real-time PCR showed that *Ba-CaM* was highly expressed in cyprids, the stage at which swimming larvae are competent to attach and undergo metamorphosis. *In situ* hybridization revealed that the expressed *Ba-CaM* gene was localized in compound eyes, posterior ganglion and cement glands, all of which may have essential functions during larval settlement. Larval settlement assays showed that both the CaM inhibitor compound 48/80 and the CaM-dependent myosin light chain kinase (MLCK) inhibitor ML-7 effectively blocked barnacle larval settlement, whereas Ca^2+^/CaM-dependent kinase II (CaMKII) inhibitors did not show any clear effects. The subsequent real-time PCR assay showed a higher expression level of *Ba-MLCK* gene in larval stages than in adults, suggesting an important role of *Ba-MLCK* gene in larval development and competency. Overall, the results suggest that CaM and CaM-dependent MLCK function during larval settlement of *B. amphitrite*.

## Introduction

Barnacles are a major sessile component in intertidal ecosystems around the world, and also a dominant hard fouler of ship hulls and other marine structures [1]. So, an economic and environmental interest has been aroused in exploring the mechanisms that govern their settlement and distributions. After six naupliar stages [2], barnacle nauplii metamorphose into a cyprid stage that is competent to attach on a suitable substratum and undergo metamorphosis into the sessile juvenile stage. During metamorphosis into the juvenile, several morphological changes occur, including the decortication of cyprid carapace, the formation of a new chitinous layer, the degeneration of compound eyes and antenna, the migration of naupliar eye, and the development of feeding cirri [3]. This transition from free-swimming larvae to sessile juveniles is crucial not only for their development, but also for species distribution and intertidal community dynamics [4].

Many previous studies have focused on the involvement of endogenous factors during barnacle larval attachment and metamorphosis (collectively known as “larval settlement”) in the cosmopolitan intertidal barnacle *Balanus amphitrite*, such as cyclic AMP [5], protein kinase C [6], settlement-inducing protein complex (SIPC) [7], and hormonal substances [8], [9], [10]. More molecular studies have been conducted in recent years to detect the differential gene and protein expression profiles during larval settlement of this species. Using a subtracted cDNA library, six genes were uniquely detected in cyprids [11] and they showed differential expression patterns during larval settlement [12]. Recently, several genes highly expressed in cyprids were isolated by comparative transcriptomic analysis and their biological functions during larval settlement were discussed [13]. Moreover, distinct proteome and phosphoproteome profiles have been identified in different developmental stages of *B. amphitrite*, with calmodulin (CaM) being identified as one of the highly expressed proteins in the cyprid stage [14], [15]. The CaM protein product first accumulated in the stage VI nauplius stage and then peaked in the cyprid stage, followed by a decline in attached larvae and juveniles [14].

CaM is a small acidic Ca^2+^-binding protein, with a structure and function that is highly conserved in all eukaryotes [16]. Upon Ca^2+^-binding, CaM interacts with several proteins called CaM-binding proteins, including phosphatases, nitric-oxide synthase, kinases, numerous receptors, transcription factors, ion channels and G protein coupled receptors [17], [18], [19]. Along with CaM-binding proteins, CaM activates various Ca^2+^-dependent enzyme reactions, thereby modulating a wide range of cellular events, including metabolism control, muscle contraction, exocytosis of hormones and neurotransmitters, and cell division and differentiation [16], [20], [21], [22]. Several CaM studies have been conducted on marine invertebrates. CaMs isolated from sea anemone and scallop muscle have a similar biological function to rabbit CaM [23]. It had also been reported that CaM is involved in the differentiation of the alimentary tract in amphioxus [24], and the development of the nervous system in ascidians [25]. Gao et al. [26] reported a link between the regulation of CaM and that of Ca^2+^ ATPase during crayfish molting. CaM has also been reported to be a pivotal calcium metabolism regulator in the shell formation of oyster [27]. In the barnacle *B. amphitrite*, low concentration of external Ca^2+^ could inhibit larval settlement [28]. As one of the Ca^2+^-binding protein, CaM was detected in both cyprids and adults of this species [29]. Using several CaM-dependent phosphodiesterase (PDE) inhibitors, Ca^2+^/CaM-dependent kinase II (CaMKII) inhibitors and myosin light chain kinase (MLCK) inhibitors, CaM was presumed to function via the enzymatic reactions of MLCK and CaMKII rather than PDE [29]. However, the detailed expression patterns of CaM and its binding proteins during larval settlement of *B. amphitrite* remain unclear.

In this study, we cloned *CaM* gene in *B. amphitrite*, analyzed its expression profiles in different developmental stages, and examined the involvement of CaM, CaMKII and MLCK in larval settlement of this species using several inhibitors. As *B. amphitrite* is one of the most widely used species to study the settlement of marine invertebrates and antifouling strategies, this study may shed light on the molecular mechanism of larval settlement and then help in antifouling studies.

## Results

### Cloning of *CaM* gene from *B. amphitrite* (*Ba-CaM*)

**Figure 1:**
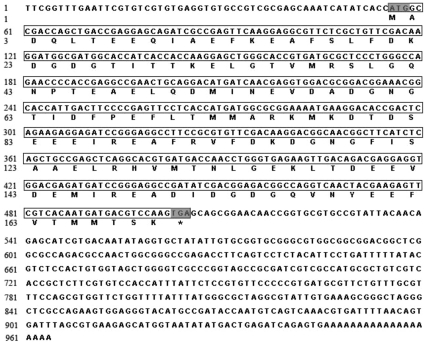
The complete nucleotide and deduced amino acid sequences of CaM in *B. amphitrite*. The nucleotides and amino acids are numbered on the left of the sequences. The sequences in the box show the whole open reading frame. An asterisk (*) represents the stop codon at the end of the coding sequence.

**Figure 2:**
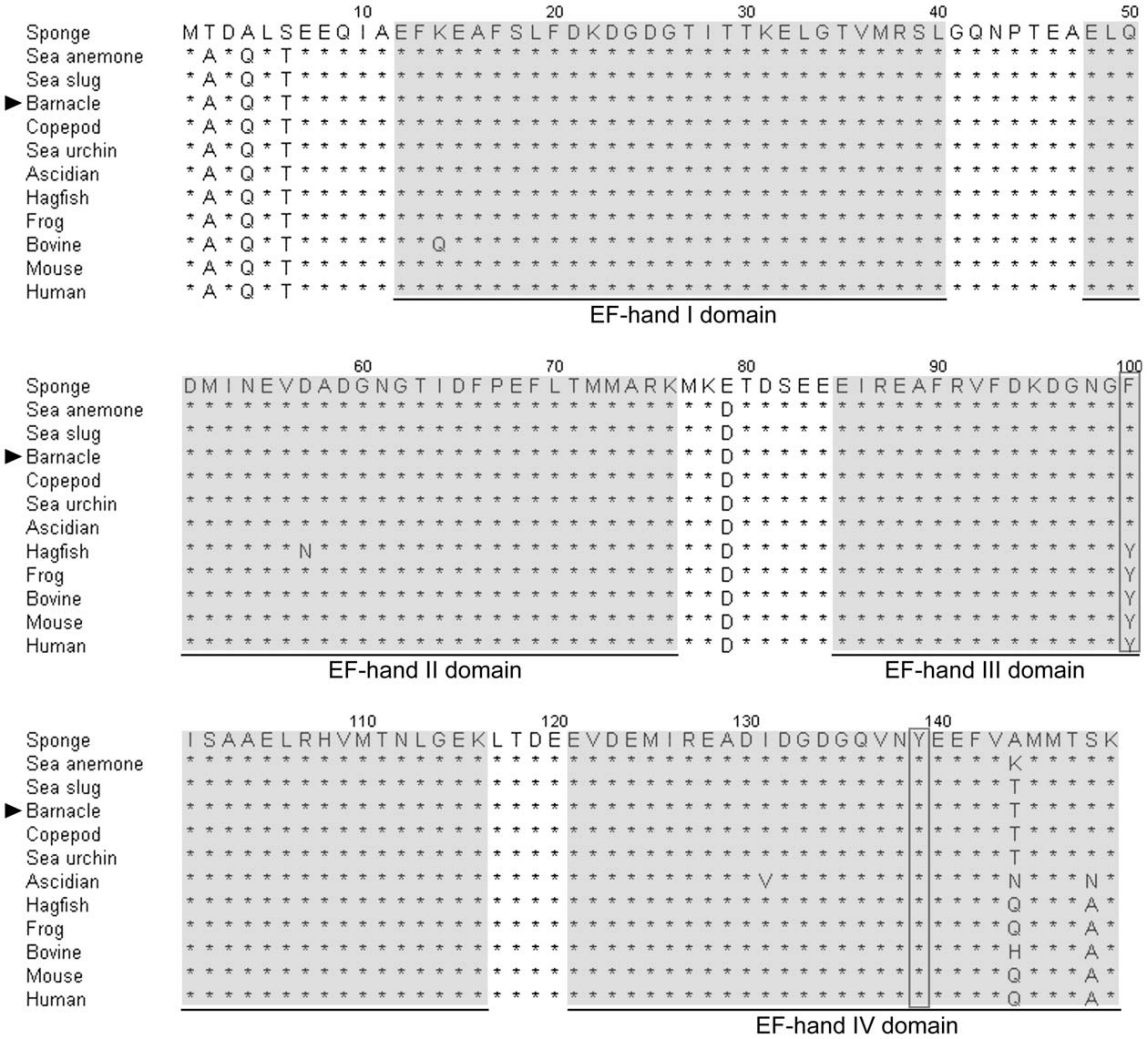
Multiple alignment of the amino acid sequence of CaM in *B. amphitrite* with its homologs in sponge (BAB61797.1), sea anemone (BAB61796.1), sea slug (NP_001191509.1), copepod (ACO10440.1), sea urchin (BAB89360.1), ascidian (NP_001027633.1), hagfish (AAD56955.1), frog (NP_001080864.1), bovine (NP_001159980.1), mouse (AAH54805.1), and human (AAD45181.1). Amino acids are numbered and asterisks (*) indicate the identical amino acids. The four EF-hand domains are underlined and the letters in boxes show the conserved tyrosine (Y) residues generally found in most invertebrates. The numbers above the sequences show the amino acid position. The shaded areas are EF-hand domains.

The full-length cDNA of *Ba-CaM* (accession no. JN314840) was 964 bp, consisting of a 5′-terminal untranslated region (UTR) of 55 bp and a 3′ UTR of 459 bp with a polyA tail (Fig. 1). The blastn result revealed that the coding region of *Ba-CaM* matched the *CaM*s from copepod (79%), sea slug (77%), ascidian (78%), sea urchin (83%), hagfish (80%), frog (77%), rat (78%), and human (74%). *Ba-CaM* gene encoded a polypeptide of 149 amino acids with a predicted molecular weight of 17 kDa. Multiple protein sequence alignment analysis (Fig. 2) showed that the CaM sequence of *B. amphitrite* shares a high similarity with the CaMs isolated from a wide range of species, indicating that CaM is a fairly conserved protein. EF-hand I-III domains were more conserved than EF-hand IV domain, which has several individual amino acids different from other CaM sequences at the *C*-terminus including Ile^131^, Thr^144^, Ser^148^. A parallel comparison of the amino acid sequences of all the EF-hands showed that EF-I is more similar to EF-III and EF-II with EF-IV. The similarities between EF-I and EF-III (55%) and EF-II and EF-IV (48%) are higher than those found in the other comparisons (31%–37%). Within four EF-hand domains, the four predicted Ca^2+^-binding sites contained amino acid residues 21–32, 57–68, 94–105 and 130–141. Similar to CaMs from most invertebrates, Ba-CaM had only one tyrosine located at the 139^th^ residue rather than dual tyrosine residues in most vertebrates (Fig. 2). Previous NMR studies have shown that the environment of the tyrosine residues changes upon Ca^2+^-binding [30].

### Copy number of *Ba-CaM*

**Figure 3:**
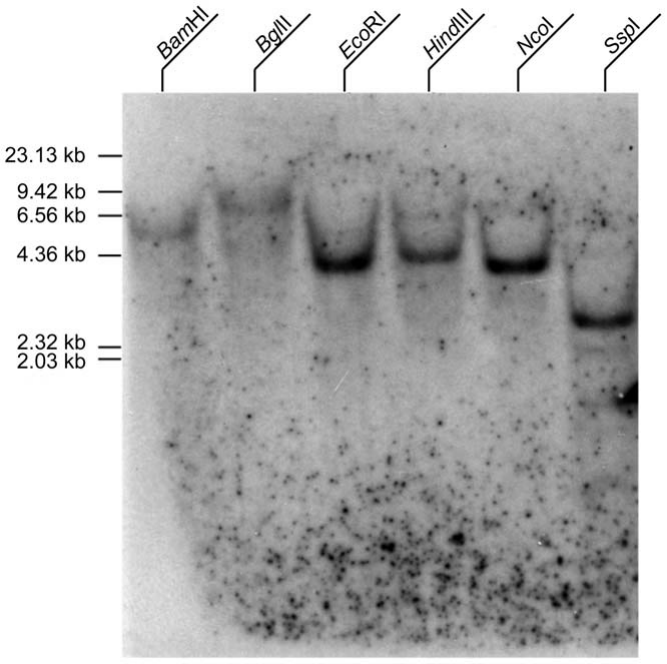
Southern blot of digested genomic DNA for the identification of the copy number of *Ba-CaM* gene. Genomic DNA was digested with various restriction enzymes including *Bam*HI, *Bgl*II, *Eco*RI, *Hind*III, *Nco*I, and *Ssp*I.

To determine the copy number of *Ba-CaM* in the genome of *B. amphitrite*, the DIG-labelled 2,300 bp fragment was prepared for Southern blot. Aligned with *Ba-CaM* cDNA sequence, this 2,300 bp fragment amplified from the genomic DNA contained two introns. Only a single band was detected in any of the genomic DNA samples individually digested with various restriction enzymes (Fig. 3), suggesting there is only a single copy of *Ba-CaM* gene in the barnacle genome.

### Gene expression pattern of *Ba-CaM*

**Figure 4:**
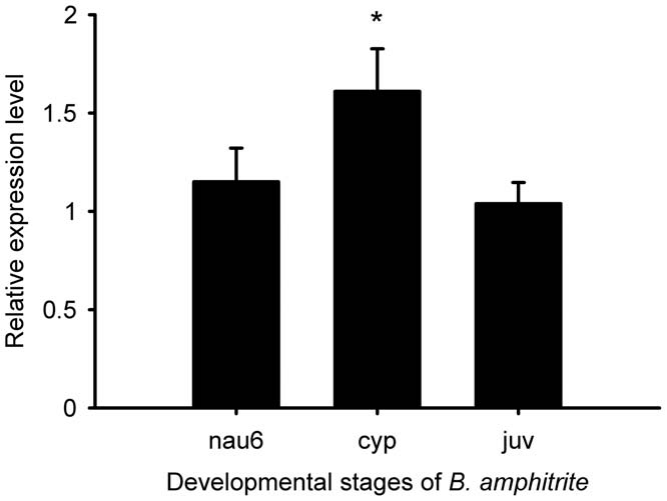
*Ba-CaM* mRNA expression levels in different developmental stages of *B. amphitrite* detected by real-time PCR. The stages included stage VI nauplius (nau6), cyprid (cyp) and juvenile (juv). Bars represent the mean ± SD (n = 3). An Asterisk (*) indicates a significant difference compared with the positive control (*P*<0.05).

**Figure 5:**
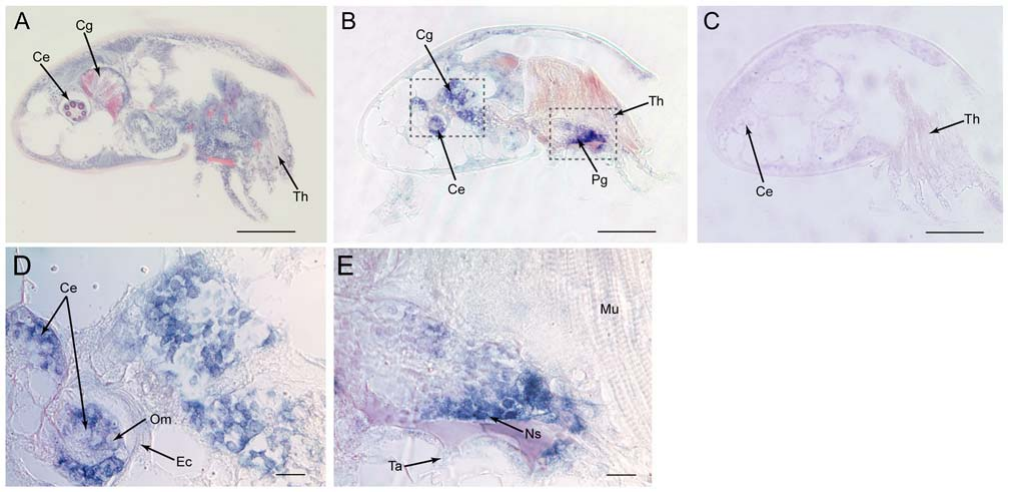
*Ba-CaM* spatial expression pattern in the sagittal sections of *B. amphitrite* cyprids. The HE staining image (A) shows a clear larval histology. Blue color stained by haematoxylin indicated nuclei, while red color by eosin indicated basic proteins or muscle fibers. Lenses of compound eyes and intracellular substances (maybe cement proteins) in the cement gland were stained by eosin. Positive signals were detected from the sections hybridized with the anti-sense probe (B). Section hybridized with the sense probe served as a control (C). (E) Detail of the cyprid compound eye and cement glands. (F) Detail of the cyprid posterior ganglion. Ce, compound eye; Cg, cement gland; Pg, posterior ganglion; Ta, thorax. Scale bars = 100 µm.

After normalization against the expression level of *Cyb* gene, *Ba-CaM* expression in stage VI nauplii was set as a calibrator to measure the relative expression levels of *Ba-CaM* in other developmental stages. The dynamic expression of the relative *Ba-CaM* mRNA was revealed by real-time PCR assays (Fig. 4). As barnacle larvae gained competency and underwent settlement, *Ba-CaM* was up-regulated until it reached a peak at the cyprid stage, and was then down-regulated in the juvenile stage. In cyprids, the expression of *Ba-CaM* was significantly increased by 1.71-fold compared with the previous stage (one-way ANOVA, *P*<0.01). However, it dropped by 1.5-fold after settlement and was not significantly different from that of stage VI nauplii (*P*>0.01). To obtain an overall picture of the expression site of *Ba-CaM* gene in *B. amphitrite*, *in situ* hybridization was performed on the sectioned cyprid tissues (Fig. 5). No signal was detected in the specimens hybridized with the sense probe (Fig. 5C), whereas a strong signal was detected in the regions of compound eyes, posterior ganglion and cement glands (Fig. 5B).

### Larval settlement assays of *B. amphitrite*

**Figure 6:**
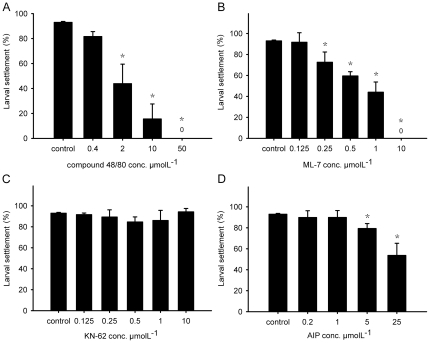
The effect of inhibitors on larval settlement of *B. amphitrite*. Inhibitor assays using (A) the CaM inhibitor compound 48/80, (B) the MLCK inhibitor ML-7, (C) the CaMKII inhibitor KN-62, and (D) the CaMKII inhibitor AIP. Data presented are the means ± SD (n = 5). Asterisks (*) indicate significant differences compared with the positive control (*P*<0.05).

The CaM inhibitor compound 48/80 effectively inhibited barnacle cyprid settlement in a dose-dependent manner (Fig. 6A). The compound had an inhibitive effect on the cyprids at concentrations ranging from 2 µM to 50 µM, but no significant effect at a concentration of 0.4 µM (*P*>0.01). To examine the target proteins that CAM might regulate, barnacle cyprids were treated with the inhibitors against two CAM-dependent protein kinases, CaMKII and MLCK, individually. ML-7, a direct inhibitor against MLCK, inhibited cyprid settlement at a concentration of 0.25 µM and a complete inhibitive effect was observed at a concentration of 10 µM (Fig. 6B). However, KN-62, one of the CaMKII inhibitors, did not show any significant effect ranging from the concentrations of 0.125 µM to 10 µM (Fig. 6C); whereas, AIP, another CaMKII inhibitor tested in this study, partially inhibited cyprid settlement at concentrations higher than 5 µM (Fig. 6D).

### Gene expression pattern of *Ba-CaMKII* and *Ba-MLCK*

A partial cDNA sequence of *Ba-MLCK* gene (accession no. JN314841) with the length of 1,626 bp was obtained, including a partial coding region followed by a polyadenylation signal sequence AATAAA and a poly (A) tail. Searching for similar sequences of *Ba-MLCK* gene using blastx analysis revealed close matches with the amino acid sequence of MLCK in fruit fly (59%), ant (58%) and mosquito (58%). The partial sequence of *Ba-CaMKII* was deposited in GenBank under accession no. JN314842.

**Figure 7:**
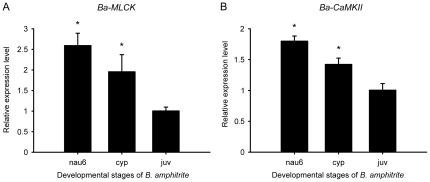
*Ba-MLCK and Ba-CaMKII* mRNA expression levels in different developmental stages of *B. amphitrite*. Relative gene expression levels of *MLCK* (A) and *CaMKII* (B) were detected by using real-time PCR in stage VI nauplius (nau6), cyprid (cyp) and juvenile (juv). Bars represent the mean ± SD (n = 3). Asterisks (*) indicate significant differences compared with the positive control (*P*<0.05).

Real-time PCR was performed to further examine the relative expression patterns of the two genes. In the three developmental stages examined, both genes were highly expressed in stage VI nauplii and then down-regulated in cyprids and juveniles (Fig. 7), but the changes were of different magnitudes. The gene expression levels of *Ba-MLCK* in stage VI nauplii and cyprids (2.81-fold and 2.24-fold, respectively) were significantly higher than that in juvenile (*P*<0.01), while the levels of *Ba-CaMKII* were 1.92-fold higher in stage VI nauplius and 1.49-fold higher in cyprid (*P*<0.01).

## Discussion

Most of the researches on CaM in crustaceans have focused on its involvement in salt transportation, pathogen response and molting process [31], [32], [33]. As barnacles have a free-swimming larval stage and sessile juvenile and adult stages, larval settlement has substantial impacts on barnacle development. Here, we described the cloning and expression patterns of *CaM* gene from the barnacle *B. amphitrite* (*Ba-CaM*), and studied the pathways in which CaM may be involved in during larval settlement.

The full-length cDNA of *Ba-CaM* gene was obtained by RACE technique. The open reading frame encodes a polypeptide of 149 amino acids with a high sequence similarity to those in other species. Multiple amino acid sequences alignment revealed that Ba-CaM is identical to the CaMs in sea anemone, sea slug, copepod and sea urchin. Moreover, conserved sequences and characteristic motifs, such as EF-hand domains and Ca^2+^-binding regions (Fig. 2), were also found in Ba-CaM amino acid sequence. The higher similarities between EF-I and EF-III or EF-II and EF-IV domain of Ba-CaM have also been reported in goldfish CaM [34], supporting the argument that CaM may have evolved from an ancestor gene encoding EF-hand with successive duplication [35]. However, although the protein structures of CaMs are conserved, the nucleotide sequences coding CaMs vary among different species. Previous studies have reported that multiple copies of *CaM* genes exist in human [36], rat [37], chicken [38], frog [39], goldfish [34] and sea urchin [40]. These *CaM* genes, can be group into three subfamilies of *CaM* I, *CaM* II, and *CaM* III [41], [42], [43], yet they encode the same CaM protein, which supports the model of “multiple genes, single protein” [41], [42]. In contrast, there is only a single copy of CaM gene in most invertebrates, such as fruit fly [44] and nematode [45]. In the barnacle *B. amphitrite*, the single band observed in the digesting genomic DNA with different restriction enzymes indicated that *Ba-CaM* occurs as a sole gene in the genome, as in many other lower invertebrates (Fig. 3).

The mRNA expression levels of *Ba-CaM* in different developmental stages, including stage VI nauplii, cyprids and juveniles, were investigated by using real-time PCR. The expression level was higher in cyprids than in stage VI nauplii and juveniles, which is consistent with the CaM protein profile reported by Thiyagarajan and Qian [14]. These results suggest that *CaM* may be important for cyprids. Among the developmental stages investigated, the cyprid stage is the most critical for attaching on a suitable place and metamorphosing into juveniles [46]. To further explore the possible role of CaM in larval settlement, the spatial expression pattern of *Ba-CaM* in cyprids was examined by *in situ* hybridization. A cluster of labeled cells was detected around the area of thorax posterior ganglion (Fig. 5E) that contains centrally positioned neuropil, fiber tracts and neuronal somata, and controls the movement of the thoracic appendages and larval swimming behavior [47]. It is possible that *Ba-CaM* is involved in the movement control of the thoracic appendages during the cyprid stage. Some labeled cells were also detected in the cement glands (Fig. 5D), suggesting a possibility that the *Ba-CaM* gene is expressed in the nerve fibers on the surface of the cement glands. Since barnacle larval settlement requires the adhesive protein secreted from the pair of cement glands [48], the function of *Ba-CaM* is likely to be related to cement protein secretion. In addition, a strong signal was observed in the region of the pair of compound eyes that consist of several visual cells and photoreceptors and are distinct from those in nauplius eyes or adult photoreceptors (Fig. 5D) [49]. However, how the two compound eyes really function and how *Ba-CaM* is really involved during larval settlement require further investigation.

To further explore the involvement of CaM during barnacle larval settlement, a specific CaM antagonist, compound 48/80 [50], was used to treat cyprids and the subsequent larval settlement response was observed. Compound 48/80 inhibited cyprid settlement at all the concentrations tested, except the lowest concentration of 0.4 µM (Fig. 6A). This inhibitory effect of compound 48/80 to CaM is similar to those of other CaM antagonists, such as W7, calmidazolium, chlorpromazine, trifluoperazine and vinpocetine [29]. To identify the possible target proteins that CaM regulates during barnacle larval settlement, the effects of CaMKII and MLCK inhibitors on larval settlement were also examined in this study. CaMKII is a serine/threonine-specific protein kinase that participates in the Ca^2+^ signal transduction to ion channels or transcriptional activators in cells [51]. Two CaMKII inhibitors, KN-62 and AIP, were used but showed different results. KN-62 had no significant inhibitory effect at any of the tested concentrations (Fig. 6C), which somehow contradicted the results reported by Yamamoto [29]. However, AIP inhibited cyprid settlement at concentrations higher than 5 µM (Fig. 6D). It has been reported that KN-62 inhibits not only CaMKII, but also other CaM-dependent protein kinases [52], [53], whereas AIP is more specific as an analog of CaMKII substrate [54]. The actual doses of the compounds within the cyprid body may be far lower than the concentrations tested [9], [29]. Further, AIP is a hydrophilic peptide, and thus, may not easily penetrate cells, leading to a high effective concentration. MLCK is another serine/threonine-specific protein kinase and is activated by the Ca^2+^/CaM complex [55]. The phosphorylation of myosin regulatory light chain by activated MLCK displays multi-functions in smooth muscle tissues as well as nonmuscle cells, such as smooth muscle contraction [56], endothelial cell retraction [57], fibroblast contraction [58], and cell shrinkage [59]. ML-7, a potent MLCK inhibitor, inhibited larval attachment and metamorphosis (Fig. 6B). At a high concentration of 10 µM, cyprids were all paralyzed with a trembling of the thorax, and at 1 µM, unattached cyprids were inclined to swim rather than walk on the surface using their antennules. From these observations, it appears that MLCK affects the muscle contraction and the motility of cyprids, so that they cannot attach on the surface of the substrate and cannot undergo metamorphosis either. The subsequent quantification of the expression of *Ba-CaMKII* and *Ba-MLCK* showed a similar trend but a different magnitude of changes (Fig. 7). The higher expression of *Ba-MLCK* gene in the swimming larval stages (including stage VI nauplii and cyprids) than that in sessile juveniles indicated a more critical biological function of this gene in larval development and competency. For instance, there are groups of muscles associated with the antennules and thorax that control larval motility and searching behavior [60], [61]. The smaller change in the expression of *Ba-CaMKII* gene from stage VI nauplii to juveniles, combined with the ambiguous effects of CaMKII inhibitor, indicated that there was no clear involvement of CaMKII during larval settlement. Based on these findings, we thus deduced that MLCK would be more important for larval settlement of *B. amphitrite*.

### Conclusions

In this study, we cloned and characterized *CaM* gene of the barnacle *Balanus amphtrite*. The gene shares conserved amino acid sequences and characteristic motifs with other CaMs in other vertebrates and invertebrates. Similar to most invertebrates, *B. amphitrite* has only one copy of *CaM* gene in its genome. *Ba-CaM* gene was highly expressed in *B. amphitrite* cyprids with the expression localized in the tissues that play essential roles in larval swimming and settlement, such as compound eyes, thoracic ganglion and cement glands. CaM and MLCK inhibitors showed distinct inhibitory effects on barnacle larval settlement. Based on these observations, we suggest that CaM and Ca^2+^/CaM dependent MLCK are crucial to larval settlement of *B. amphitrite*.

## Materials and Methods

### Animals

The barnacle, *Balanus amphitrite*, that we used for this study is a common species of marine invertebrates. It is a biofouling species and not endangered or protected. The adults of *B. amphitrite* were collected from a dock at Pak Sha Wan, Hong Kong (22.21′45″N, 114.15′35″E). No specific permits were required for the described field studies. The dock does not belong to any national parks, protected areas, or privately-owned places. The filed studies did not involve any endangered or protected species.

Larvae from different developmental stages were obtained and cultured as described by Thiyagarajan and Qian [14]. Nauplii were released from the adults and cultured at 27°C on a diet of *Chaetoceros gracilis* Schutt for four days (through six molts) until the larvae molted into cyprids.

### Total RNA extraction

Stage II nauplii (within 2 hr after released from the adults), stage VI nauplii (day 3 larvae with compound eyes), cyprids (day 4 larvae attaining competency to settle) and young juveniles (24 hr post-settlement) were collected and stored in liquid nitrogen until use. Total RNA was isolated from each sample by using TRIzol reagent (Invitrogen, Carlsbad, CA, USA) according to the manufacturer's protocol. The quantity and quality of RNA was checked with agarose gel electrophoresis and a NanoDrop 1000 spectrophotometer (Thermo Fisher Scientific, Waltham, MA, USA). After treatment with Turbo DNAfree kit (Ambion Inc, Austin, TX) to remove the trace genomic DNA contamination, the cDNA was synthesized with oligo dT_(18)_ primer using MMLV reverse transcriptase (USB, Cleveland, OH).

### Cloning of the genes from *B. amphitrite*

Barnacle *CaM* gene (*Ba-CaM*) was isolated by degenate PCR and the fragments of barnacle *MLCK* gene (*Ba-MLCK*) was obtained from the customized transcriptome database. In order to amplify the cDNA fragment of *Ba-CaM*, two sets of nested degenerate primers were designed based on the CaM amino acid sequences from fruit fly (GenBank accession no. P62152), zebrafish (Q6PI52), sea squirt (CAA73906), scallop (P02595), and nematode (O16305), 5′-ACNGARGARCARATYGCWGA-3′ (SDF1), 5′-ATCATBKKYACRAAYTCTTC-3′ (SDR1), 5′-AYGGSGAYGGNACMATMACMAC-3′ (SDF2) and 5′-TCHCCRTCRAYRTCWGCTTCYC-3′ (SDR2), which targeted conservative regions predicted by alignment (using ClustalW) of the CaM amino acid sequences. Degenerate PCR was carried out using the following protocol with a hot start: 94°C for 5 min, 32 cycles of 94°C for 45 sec, 52°C for 15 sec, and 72°C for 1 min, and followed by a final extension at 72°C for 10 min. Afterwards, based on the fragment of each gene, four gene-specific primers were designed for the rapid amplification of cDNA ends (RACE) reaction of individual gene. The primers for *Ba-CaM* are, 5′-ACTTCCCCGAGTTCCTCACCA-3′ (CaMGSF1), 5′- AGGACGGCAACGGCTTCA-3′ (CaMGSF2), 5′-TTCTCACCCAGGTTGGTCATCA-3′ (CaMGSR1), 5′-ATGAAGCCGTTGCCGTCCT-3′ (CaMGSR2). The primers for *Ba-MLCK* are, 5′-ACGACTTCACGCTGACGGA-3′ (MLCKGSF1), 5′- TGCGCTACATGCACGACAACA-3′ (MLCKGSF2), 5′-TGACGCCCAGACTCCACAT-3′ (MLCKGSR1), 5′-ACTGATGGGCTCGAAGTTGATGAT-3′ (MLCKGSR2). Nested PCR was carried out to amplify the 3′ terminus with GSF1 primers, oligo dA_(18)_+adaptor primer and GSF2 primers, the RACE adaptor primer. For the 5′ RACE, oligo dC tail was added to the 3′ cDNA end with terminal deoxynucletidyl tranferase (USB) reaction. The 5′ terminus were then amplified using nested PCR with GSR1 primers, oligo dG_(10)_+adaptor primer and GSR2 primers, the RACE adaptor primer. The amplification program was similar to that for the degenerate PCR, but with an annealing temperature of 58°C. All the PCR products were purified and ligated with pMD18-simple T vectors (Takara, Dalian, China). The transformations were performed according to the standard protocol. Positive clones were screened with PCR and then sent for sequencing.

### Southern blot hybridization

Genomic DNA was extracted from the homogenized cyprids in an extraction buffer containing 2% SDS, 0.3 M NaCl, 5 mM EDTA (pH 8.0), 50 mM Tris-HCl (pH 8.0), 0.4 mg/mL RNase and 0.5 mg/mL proteinase K. After extraction with phenol-chloroform (24∶1, vol/vol), DNA was precipitated with 1 vol. of isopropanol and 1/10 vol. of 5 M NH_4_OAC, and then washed twice with 75% ethanol. DNA (30 µg) from each sample was digested with restriction enzymes *Bam*HI, *Bgl*II, *Eco*RI, *Hind*III, *Nco*I and *Ssp*I, respectively, After incubation at 37°C overnight, the digested DNAs were analyzed on a 0.8% agarose gel and transferred to a nylon membrane (NB0HY00010, GE, Minnetonka, MN) using the capillary transfer method. A *Ba-CaM* probe was labeled with biotin using Brightstar Psoralen-Biotin labeling kit (Ambion Inc, Austin, TX) and hybridization was performed at 42°C following GE nylon membrane operation protocol. Detection was carried out with Brightstar BioDetect kit (Ambion).

### Real-time PCR assay

The template cDNAs from different developmental stages were synthesized from 2 µg of total RNA from each stage. Based on the sequence information obtained from cloning, two primers, 5′-CGTTCTGTTTGCGTTTCCA-3′ (CaMRTF), 5′-TGACTGACATTGGTATCGGCAT -3′ (CaMRTR), were designed for a 116 bp amplicon for *Ba-CaM* gene expression analysis. Based on the customized transcriptome database, two pairs of primers were designed for the amplification of barnacle *CaMKII* (*Ba-CaMKII*) and *Ba-MLCK* genes. The primers were: 5′- ACAACGTGTTGGGCAAGTTCCG-3′ (CaMKIIRTF), 5′- TCAATATACTGCGAGAGCCGCA-3′ (CaMKIIRTR), 5′- TGGGCGATAACGACGCCGAAAC-3′ (MLCKRTF), and 5′- CTCATCCGCTCCGTTTGTCGCT-3′ (MLCKRTR). Cytochrome b (*Cyb*) was chosen as the reference gene for internal standardization. Two *Cyb* primers, 5′-GGACACTGCATGCTAATGGA-3′ (CybF) and 5′-AGGCAGCAGCCATAGTCAAG-3′ (CybR), were used to amplify a *Cyb* gene fragment of 144 bp [62]. The assays were performed on Stratagene Mx3000P QPCR System (Agilent, Santa Clara, CA) in a final volume of 10 µL containing 5 µL SYBR Green Supermix (BioRad, Hercules, CA), 1 µL of 10-fold diluted cDNA as the template, 0.4 µL of each of the primers and 3.2 µL PCR-grade water. The amplification program was as follows: 3 min at 95°C for one cycle, followed by 40 cycles of 30 sec at 95°C, 40 sec at 58°C, and 1 min at 72°C. Dissociation curve analysis of the PCR products at the end of each reaction ensured the specificity of the amplifications. The cDNA sample was analyzed in experimental triplicates and the relative fold change was calculated based on the relative quantification ΔΔCt method. The data were subjected to a Student's *t*-test and significance was accepted at *P*<0.05.

### *In situ* hybridization (ISH) of tissue sections of *B. amphitrite*

The *B. amphitrite* cyprids were collected, relaxed in 0.37 M MgCl_2_ with autoclaved filtered seawater (1∶1) for 10 min, and then fixed with freshly prepared fixative (0.5% glutaraldehyde, 4% paraformaldehyde in phosphate-buffered saline, pH 7.4) at 4°C overnight. Larvae were dehydrated in a successively graded ethanol series (25%, 50%, 75%, and finally 100%), embedded in paraffin wax (Paraplast Plus, Kendall), cut into 7-µm sections, and mounted on Poly-L-lysine coated slides.

The presence of *Ba-CaM* mRNA transcript was detected using digoxigenin (DIG) labeled antisense and sense RNA probes (DIG RNA labeling kit, Roche Diagnostics, Nutley, NJ), which were synthesized from a 791 bp PCR product by a pair of gene-specific primers (5′- TCAAGGAGGCGTTCTCGCTGTT-3′, CaMISHF; 5′- TTGACTGACATTGGTATCGGCAT-3′, CaMISHR). The T7 RNA polymerase promoter (5′ TAATACGACTCACTATAGGG-3′) was included in the appropriate primers for sense/antisense probe synthesis [63]. The *in situ* hybridization protocol was performed according to Dreanno et al. [64] with minor modifications. After dewaxed in xylene and rehydrated in a successive dilution of ethanol, the slides were rinsed in 0.2% glycine in PBS twice for 5 min each, then incubated in 10 µg/mL Proteinase K in PBS for 4 min at 37°C. The samples were post-fixed in 0.5% glutaraldehyde, 4% paraformaldehyde in PBS for 20 min and then washed three times for 5 min each in 0.1% Tween-20 in PBS (PBT). After pre-hybridization for 2 hr at 65°C in a hybridization mix (50% formamide, 5×SSC, 1% SDS, 0.1% Tween 20, 50 µg/mL heparin, 100 µg/mL salmon sperm DNA), the specimens were hybridized with fresh hybridization mix containing 0.5 µg/mL Dig-labeled RNA probe at 65°C overnight. The slides were incubated in 50% formamide, TNE buffer (10 mM Tris-HCl, pH 7.6, 1 mM EDTA, 500 mM NaCl) at 65°C and then, in RNase (10 µg/µL) in TNE buffer at 37°C for another 30 min. The washing steps were performed with 2× SSC and 0.2× SSC at 65°C for 20 min separately, followed by washing in DIG buffer (100 mM Tris-HCl, pH 7.6, 150 mM NaCl) for 5 min at room temperature. The samples were blocked in a blocking buffer (4% BSA and 2% goat serum in DIG buffer) at room temperature for 2 hr and incubated in 1∶5000 alkaline phosphatase (AP)-conjugated anti DIG at 4°C overnight. The signal was developed in the dark overnight using nitroblue tetrazolium (NBT; Promega) and 5-bromo-4-chloro-3-indolylphosphate (BCIP; Promega) diluted in alkaline Tris buffer (100 mM Tris HCl, pH 9.5, 50 mM MgCl_2_, 100 mM NaCl and 0.1% Tween 20 (vol/vol)), then the reactions were terminated with a stop solution (1 mM EDTA in PBT). The slides were dehydrated and mounted with DPX mountant (Fluka, Buchs, Switzerland) for histology.

### Larval settlement assay

Fresh cyprids were obtained and treated with four compounds, including the CaM inhibitor Compound 48/80 (Alexis, Lausen, Switzerland), the CaMKII inhibitor KN-62 (Sigma-Aldrich, St Louis, MO), the CaMKII inhibitor AIP (Alexis, Lausen, Switzerland), and the MLCK inhibitor ML-7 (Biomol, Plymouth, UK) in multi-well polystyrene plates (Nunc, Roskilde, Denmark). Stock solutions (100 mM for KN-62, ML-7 and compound 48/80, and 25 mM for AIP) were prepared by dissolving each in either dimethyl sulfoxide (KN-62 and ML-7) or water (compound 48/80 and AIP) and then stored at −20°C. One milliliters of filtered seawater (FSW) was added to each well, into which 12±2 cyprids were placed. The plates were then placed in dark at 22°C until the settled and swimming larvae were counted under a dissecting microscope. Cyprids in FSW without any compounds were be used as positive controls. Each treatment was conducted in five replicates (n = 5). Significant differences among the treatments and the controls were detected using one-way ANOVA followed by a two-sided Dunnett's post hoc test.
